# Runners with a high body mass index and previous running‐related problems is a high‐risk population for sustaining a new running‐related injury: A 18‐month cohort study

**DOI:** 10.1002/ejsc.12206

**Published:** 2024-12-10

**Authors:** Ida Lindman, Josefin Abrahamson, Rasmus O. Nielsen

**Affiliations:** ^1^ General Practice / Family Medicine School of Public Health and Community Medicine Institute of Medicine Sahlgrenska Academy University of Gothenburg Gothenburg Sweden; ^2^ Research, Education, Development & Innovation Primary Health Care Region Västra Götaland Sweden; ^3^ Orthopaedic Research Unit Sahlgrenska University Hospital Gothenburg Sweden; ^4^ Department of Health and Rehabilitation Institute of Neuroscience and Physiology at Sahlgrenska Academy University of Gothenburg Gothenburg Sweden; ^5^ Department of Public Health Aarhus University Aarhus Denmark; ^6^ Research Unit for General Practice Aarhus Denmark

**Keywords:** body mass index, running, running‐related injury

## Abstract

High body mass index (BMI) and a previous running‐related injury (RRI) have been highlighted as two risk factors for sustaining an RRI. However, a critical gap exists in the knowledge of whether runners with both elevated BMI and a previous RRI constitute a particularly vulnerable subgroup in terms of susceptibility to new RRIs. Therefore, the present study aimed to evaluate if those with high BMI and a concomitant history of running‐related problems in the past 3 months were more prone to sustain a new RRI compared with runners with normal BMI and without previous running‐related problems. This study was part of the “Garmin‐RUNSAFE Running Health Study,” an 18‐month cohort study. The runners completed a baseline questionnaire containing questions regarding demographic data and previous running‐related problems and were asked to continuously track their running activities. The exposure were dichotomized into “no previous running‐related problem” or “previous running‐related problem,” and each group was further categorized into four subgroups depending on BMI. Time‐to‐event analysis was used to estimate the cumulative incidence risk difference (cIRD). The results highlight those with a BMI >30 kg/m^2^ *with* a previous running‐related problem to face the highest injury risk of 71%, whereas those with a BMI between 19 and 25 kg/m^2^ *without* a previous running‐related problem had the lowest injury risk of 43% corresponding to a cIRD of 28% [95% CI: 19%; 36%]. This result highlights those with high BMI and previous running‐related problems as a high‐risk subpopulation that would benefit from interventions of preventing running‐related injuries.

## Highlights

- Runners with previous running‐related problems and a concomitant high body mass index (BMI) have a higher risk of a new running‐related injury compared with runners with normal BMI without a history of running‐related problems.
- This finding suggests that runners with high BMI and previous running‐related problems would benefit from interventions that prevent running‐related injuries.

## 1 INTRODUCTION

Running has emerged as one of the most popular forms of physical activity worldwide, embraced by individuals of different ages, backgrounds, and fitness levels. Its appeal lies not only in its accessibility but also in its potential to improve cardiovascular health, mental well‐being, and overall fitness (Hespanhol Junior et al., 2015; Lee et al., 2011; Pedisic et al., 2020). As a result, people engage in running to stay active, with participation spanning from recreational joggers to competitive athletes. This contributes to its status as a ubiquitous form of exercise across the globe.

Despite its reported health‐related benefits, running is not without risks. Running‐related injuries (RRI) represent a concern among athletes, affecting individuals across various running disciplines and experience levels (Nielsen et al., 2024; Videbæk et al., 2015). A meta‐analysis revealed that the incidence of RRI ranges from 2.5 to 33.0 injuries per 1000 h of running, depending on the level of the runner and the definition of RRI (Videbæk et al., 2015). These incidence rates of RRI underscore the importance of understanding predictors of injury and allowing runners, coaches, clinicians, and researchers to better understand which type of runners that are particularly vulnerable toward sustaining an RRI.

Among the established predictors for RRI, high body mass index (BMI) and previous injury history have gained significant attention within the scientific community (Benca et al., 2020; Bertelsen et al., 2018b; Buist et al., 2010; Desai et al., 2021; Kluitenberg et al., 2015; Nielsen, Bertelsen, et al., 2014). Higher BMI has been associated with increased mechanical stress on weight‐bearing joints, potentially predisposing individuals to musculoskeletal injuries during running activities (Bowser et al., 2021; Vincent et al., 2020). Due to their increased body weight, there is a greater load per step, meaning that shorter time to reach a cumulative load equal that of a runner with normal BMI (Bertelsen et al., 2018a; Vincent et al., 2020). Similarly, a history of previous RRI has been highlighted as one of the strongest predictors to future injuries (Burke et al., 2023; Desai et al., 2021; van et al., 2007; van et al., 2021). Despite the wealth of research exploring the individual predictions of BMI and previous injury on RRI risk, limited attention has been directed toward examining the two exposures combined within the same cohort of runners. Consequently, a critical gap exists in the understanding of whether individuals with both high BMI levels and prior RRI constitute a particularly vulnerable subgroup in terms of susceptibility to a new RRI. Addressing this knowledge gap is paramount for better understanding this subgroup of runners, potentially benefitting from targeted injury prevention strategies and optimizing training protocols to safeguard the well‐being of these runners.

The purpose of this cohort study was to investigate if those with high BMI and a concomitant history of running‐related problems in the past 3 months represents as a subgroup of runners that sustain more RRIs compared with runners with lower BMI and/or no previous running‐related problems.

## 2 MATERIALS AND METHODS

### 2.1 Study design

This study was part of the prospective cohort study entitled the Garmin‐RUNSAFE Running Health Study (Nielsen et al., 2019). This study was an 18‐month cohort study, aiming to investigate if a dose‐response relationship between changes in training‐load and the occurrence of RRI exists and subsequent how risk of RRI is associated with other variables. The Danish Data Protection Agency approved the study (reference number 2015‐57‐0002). No permission from the system of research ethics committees was needed, in accordance with the Danish Act on Research of Health Projects, Section 14, no. 2, as it falls under the category of an observational study. Furthermore, the Strengthening the Reporting of Observational studies of Epidemiology checklist was followed (Cuschieri, 2019).

### 2.2 Study population

Runners familiar with the English language and using a Garmin wearable device in tracking their training were included from all around the world. Runners at all levels were welcome to participate. The recruitment of runners took place between July 4 and December 1, 2019. The follow‐up extended from August 2019 until January 2021; hence, the length of follow‐up period varied up to 18 months between participants. An online informed consent form was signed by all included runners.

The inclusion criteria were as follows: (1) runners >18 years old. (2) The use of a Garmin wearable device and provide the running data via the Garmin Connect app. Runners were not eligible to participate if multiple people used the same Garmin profile.

### 2.3 Outcome

Every Sunday, a questionnaire including injury status was automatically e‐mailed to the runners. The runners classified themselves into 1 of 3 categories: (1) injury‐free, (2) uninjured, yet with problems (i.e., new problems or same problems as reported last week), or (3) injured, by the question “*In the past week, have you had a musculoskeletal injury or have you experienced a problem to muscles, tendons, or bones that is fully or partly caused by running?*.” An injury was defined as something painful and irritating, leading to a reduction in running activity (i.e., volume, intensity, and frequency), whereas a running‐related problem was defined as less severe than an injury, painful and irritating, yet where the runners were able to continue their running activity. This approach was based on the running injury consensus defined by Yamato et al. (2015) and the Oslo Trauma Research Center questionnaire (Clarsen et al., 2013). In addition, the runners were required to upload all their data from running sessions to Garmin Connect (a worldwide web‐based training dairy; https://connect.garmin.com/) during follow‐up.

### 2.4 Exposures

The baseline questionnaire, including self‐reported information regarding demographics, height, weight, and previous running‐related problem, was completed at inclusion. Of these, BMI and previous running‐related problems 3 months prior to baseline were the exposures/predictors of interest. The runners were categorized into four exposure groups based on their BMI: < 19 kg/m^2^ (underweight), between 19 and 25 kg/m^2^ (normal weight), between 25 and 30 kg/m^2^ (overweight), and >30 kg/m^2^ (obese).

Previous running‐related problems were based on the question “*Have you had any running‐related problems during the PAST THREE MONTHS? (pain, ache, stiffness, swelling, instability/giving way, locking, or other complaints that affect any of your normal running activities)*” to which the runners had two response options: “Yes, I have” or “No, I have not.” Based on these two predictor variables, eight exposure groups were defined: (i) BMI < 19 kg/m^2^ *without* any previous running‐related problem, (ii) BMI < 19 kg/m^2^ *with* a previous running‐related problem, (iii) BMI between 19 and 25 kg/m^2^ *without* any previous running‐related problem (reference group); (iv) BMI between 19 and 25 kg/m^2^ *with* a previous running‐related problem, (v) BMI between 25 and 30 kg/m^2^ *without* any previous running‐related problem, (vi) BMI between 25 and 30 kg/m^2^ *with* a previous running‐related problem, (vii) BMI > 30 kg/m^2^ *without* any previous running‐related problem, and (viii) BMI > 30 kg/m^2^ *with* a previous running‐related problem.

### 2.5 Statistics

Time‐to‐event analysis (a generalized linear model using the pseudo‐observation method) was used to estimate the cumulative incidence risk difference. By this method, the cumulative injury proportion is not merely a result of dividing the number of injuries by the runners at risk, since kilometer (km) to injury or censoring is incorporated into the equation (Jungmalm et al., 2020). Runners were censored if they ceased uploading their running sessions during follow‐up, stopped answering the weekly questionnaires during four consecutive weeks, were unwilling to continue in the study, or completed the follow‐up on December 31, 2020, whichever came first. By using the concept of censoring, the runners were included in the analysis although they did not complete the entire follow‐up, whereas those that were excluded were not included in the analysis. The risk difference (RD) was determined by subtracting the cumulative incidence proportion with censoring (CIP‐C) value from the reference group. Confidence interval (CI) and *p* values were computed using robust variance estimation to account for nonnormality of the pseudo‐observations. To model cumulative RD, the id option in STATA (version 18, Texas, USA) was used (Nygård Johansen et al., 2020). Kaplan–Meier survivor plot was performed with the time point 1000 km chosen for the main analysis. Kilometers were used as timescale as reported in other studies (Nielsen, Bertelsen, et al., 2014; Nielsen, Parner, et al., 2014). Since the time point of 1000 km was selected without scientific justification and other time point could have been used, the Kaplan–Meier graph allows estimation of the proportion of injury‐free runners and compare the differences between the eight exposure groups at other time points as well.

## 3 RESULTS

**FIGURE 1:**
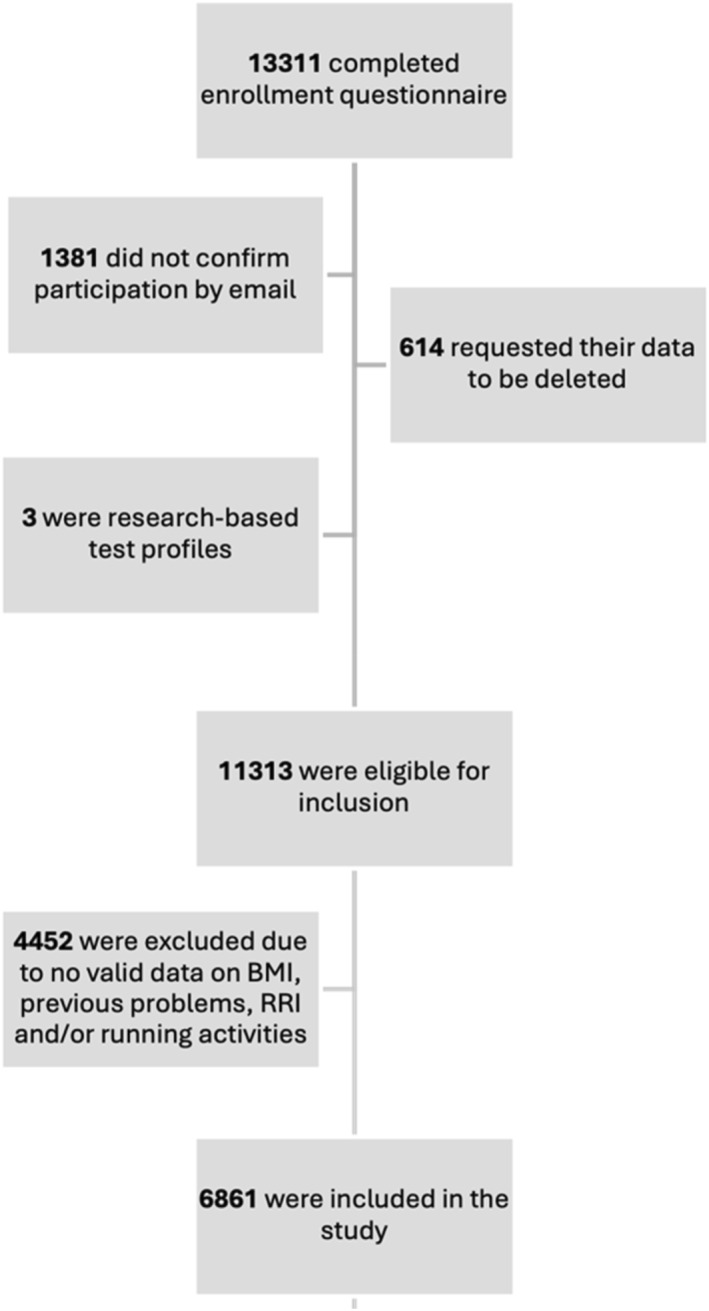
Flowchart of included runners. Of the 11,313 runners eligible for inclusion, 4452 were excluded and 6861 were included. BMI, body mass index and RRI, running related injury.

In total, 13,311 runners volunteered for the Garmin‐RUNSAFE Running Health Study, of which 4452 were excluded and 6861 fulfilled the inclusion criteria for the present study (Figure 1). Mean BMI was 24.3 kg/m^2^ and a total of 3684 (54%) runners had a history of previous running‐related problems 3 months prior to baseline. Table 1 displays the demographic data.

**TABLE 1:** Demographic data presented as mean (SE) or numbers (%).

Variable	Number = 6861
Sex	
Male, *n* (%)	5308 (77.4)
Female, *n* (%)	1553 (22.6)
Age, mean (SE)	46 (0.13)
Height, cm (SE)	176.0 (0.11)
Weight, kg (SE)	75.4 (0.16)
BMI, kg/m^2^	24.3 (0.04)
Previous running‐related problem	
Yes, *n* (%)	3684 (54)
No, *n* (%)	3177 (46)
Continent, *n* (%)	
Europe	3813 (55.6)
North America	2824 (41.2)
Africa	130 (1.9)
Asia	50 (0.7)
South America	29 (0.4)
Oceania	15 (0.2)

Abbreviations: BMI, body mass index; cm, centimeter; kg, kilogram; km, kilometers; min, minutes; *n*, numbers; and SE, standard error.

**FIGURE 2:**
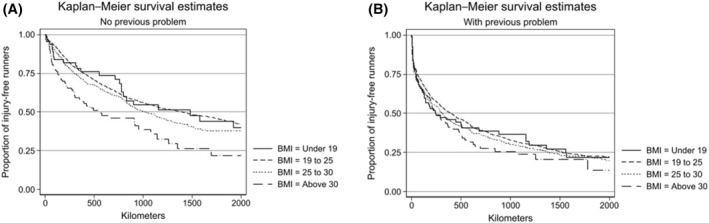
Kaplan–Meier graphs for the proportion of injury‐free runners using kilometers as timescale on the *x*‐axis. (A) Displays the runners without a previous running‐related problem, whereas (B) displays the runners with a previous running‐related problem.

In Table 2, the proportion that sustained an injury up to 1000 km is presented for each exposure group. The results highlight those with a BMI >30 kg/m^2^ *with* a previous running‐related problem to face the highest injury risk of 71%, whereas those with a BMI between 19 and 25 kg/m^2^ *without* a previous running‐related problem had the lowest injury risk of 43%. Figure 2 further displays the proportion of injury‐free runners as a function of kilometers divided into subgroups and compared between previous running‐related problems or no previous running‐related problems.

**TABLE 2:** Proportion of runners sustaining a running‐related injury among 6861 runners with different BMI and different history of running‐related problems in the 3 months preceding baseline.

BMI	No, previous running‐related problem			Yes, previous running‐related problem		
	*n*	CIP (%)	RD [95% CI]	*n*	CIP (%)	RD [95% CI]
<19	69	44.7	1.3% [−13.8%; 16.5%]	92	63.7	20.3% [7.8%; 32.9%]*
19–25	2100	43.4	Reference group	2302	67.4	24.0% [20.4%; 27.6%]*
25–30	855	49.4	6.0% [0.9%; 11.0%]*	1085	69.3	26.0% [21.6%; 30.4%]*
>30	153	60.0	16.6% [6.3%; 26.9%]*	205	71.0	27.6% [19.0%; 36.2%]*

Abbreviations: BMI, body mass index; CI, confidence interval in percentage; CIP, cumulative incidence proportion at 1000 km; and RD,  risk difference, which is the difference in percent‐point compared with the reference group (those with a BMI between 19 and 25, which did not report an running‐related problem in the 3 months preceding baseline).

* Statistical difference at a *p*‐value <0.01.

## 4 DISCUSSION

The main finding of this study was that the high‐risk population for a new RRI is the runners with the combination of high BMI and previous running‐related problem. Runners with a previous running‐related problem and a BMI >30 had a 71% risk of facing an RRI compared with runners without a previous running‐related problem and normal BMI who faced a risk of 43%, with an RD of 27.6% (CI 19%–36.2% and *p* < 0.01). There are several studies presenting BMI and previous RRI as individual predictors of sustaining a new RRI (Benca et al., 2020; Kluitenberg et al., 2015). However, this study is unique in its way of combining these two factors and showing that there is a high risk of sustaining a new injury if possessing both a high BMI and a previous running‐related problem.

It has been highlighted that the etiology of RRI is multifactorial, with the fundamental cause of excessively load in relation to capacity (Bertelsen et al., 2017, 2018b). Extensive cumulative load has been suggested as one of the main reasons for increased risk of RRI for runners with higher BMI (Bertelsen et al., 2018a, 2018b). However, the reason for the increased risk for runners with a previous RRI has not been fully understood. Insufficient recovery from previous injury, acquiring new running techniques to prevent previous pain, and a too steep start when returning to running are potential factors contributing to this elevated risk (Desai et al., 2021; Hespanhol Junior et al., 2013). As shown in this study, runners with high BMI and previous running‐related problems is a high‐risk population of sustaining a new RRI. It could be speculated that this population cannot be exposed to the same load compared with those with a normal BMI.

A high BMI is considered a risk factor for several health issues including the metabolic syndrome, cardiovascular health, and cancer. On the opposite, physical activity has been shown to reduce the risk regardless of BMI (Ahmadi et al., 2022; Herman et al., 2012). Hence, there are several positive effects for exercising with a high BMI, both its direct effects on health as well as weight loss with further health benefits (Hespanhol Junior et al., 2015). The reasons for running in this study were mainly to sustain health (see Table S1); hence, it is crucial to encourage participation in running. However, the occurrence of an RRI is a substantial reason for quitting with running, thus preventive strategies and well‐balanced running programs are advocated, and especially for this population, in order to promote public health.

Furthermore, a previous randomized controlled trial showed fewer RRIs among obese runners following a running program with reduced distance (Bertelsen et al., 2018b). Despite this, when choosing a starting program, overweight runners accomplished a similar amount of running as their normal‐weight counterparts (Bertelsen et al., 2018a). These previous studies suggest that in order to decrease the risk of RRI, a reduced running‐program may be beneficial for runners with increased BMI. However, it is important to consider other subgroups within the cohort of overweight runners, potentially more prone to RRI. Not all runners with a high BMI sustain an RRI; hence, other additional factors, such as a previous RRI, can explain why some runners are more vulnerable for an RRI. General restrictive recommendations for overweight runners may instead inversely interfere with the adherence to running. Hence, the results of the current study suggest additional caution for obese runners with a previous running‐related problem. Therefore, it should be recommended that obese runners with a previous injury or problems pay extra attention and time in rehabilitation and further use a reduced running‐program for a longer period of time after an RRI.

There are several strengths to this study. The prospective cohort study design, with a large number of runners, including different running‐levels and different parts of the world increase the generalizability of the study. The analytical aspect with censoring in the calculation of cumulative incidence is a further strength. It is unique in its way of comparing a concomitant high BMI and previous running‐related problems. However, there are limitations needed to be addressed. There is an inherited risk with self‐reported data, although the prospective design decreases the risk of recall bias. Furthermore, the vast majority of the included runners were from Europe and North America, which influences the generalizability of the study. There was a clear definition of injury; however, there was no prerequisite that the injuries were examined or diagnosed by a physician or a physiotherapist. However, the main purpose was to evaluate if high BMI and previous running‐related problems increased the risk of receiving an RRI, and not to report on the site of injury, as this has been described extensively previously. Another limitation of the study is the static categorization of participants into baseline exposure groups based on BMI and previous injury status, not accounting for potential multistate transitions, such as participants moving between BMI categories due to weight changes or transitioning from a previously injured to a noninjured state over time. This static classification could lead to misclassification and hence information bias, particularly if changes in exposure status occur during the follow‐up period that are relevant to injury risk. Despite this limitation, our findings still provide valuable insights into the risk associated with baseline characteristics, which was the primary aim of the study.

## 5 CONCLUSION

Runners with previous running‐related problems and a concomitant high BMI have a high risk of a new RRI compared with runners with normal BMI without a history of a previous running‐related problem.

## CONFLICT OF INTEREST STATEMENT

The coauthors declare that they have no conflicts of interest.

## PATIENT CONSENT STATEMENT

An online informed consent form was signed by all included runners.

## Supporting information

Table S1

## References

[ejsc12206-bib-0001] Ahmadi, M. N. , I. M. Lee , M. Hamer , B. del Pozo Cruz , L. J. Chen , E. Eroglu , Y.‐J. Lai , P. W. Ku , and E. Stamatakis . 2022. “Changes in Physical Activity and Adiposity with All‐Cause, Cardiovascular Disease, and Cancer Mortality.” International Journal of Obesity 46(10): 1849–1858. 10.1038/s41366-022-01195-z. 35915134 PMC9492547

[ejsc12206-bib-0002] Benca, E. , S. Listabarth , F. K. J. Flock , E. Pablik , C. Fischer , S. M. Walzer , R. Dorotka , R. Windhager , and P. Ziai . 2020. “Analysis of Running‐Related Injuries: The Vienna Study.” Journal of Clinical Medicine 9(2): 438. 10.3390/jcm9020438. 32041127 PMC7073658

[ejsc12206-bib-0003] Bertelsen, M. L. , M. Hansen , S. Rasmussen , and R. O. Nielsen . 2018a. “How Do Novice Runners with Different Body Mass Indexes Begin a Self‐chosen Running Regime?” Journal of Orthopaedic & Sports Physical Therapy 48(11): 873–877. 10.2519/jospt.2018.8169. 29932876

[ejsc12206-bib-0004] Bertelsen, M. L. , M. Hansen , S. Rasmussen , and R. O. Nielsen . 2018b. “The Start‐To‐Run Distance and Running‐Related Injury among Obese Novice Runners: A Randomized Trial.” International Journal of Sports Physical Therapy 13(6): 943–955. 10.26603/ijspt20180943. 30534460 PMC6253747

[ejsc12206-bib-0005] Bertelsen, M. L. , A. Hulme , J. Petersen , R. K. Brund , H. Sørensen , C. F. Finch , E. T. Parner , and R. O. Nielsen . 2017. “A Framework for the Etiology of Running‐Related Injuries.” Scandinavian Journal of Medicine & Science in Sports 27(11): 1170–1180. 10.1111/sms.12883. 28329441

[ejsc12206-bib-0006] Bowser, B. J. , and K. Roles . 2021. “Effects of Overweight and Obesity on Running Mechanics in Children.” Medicine & Science in Sports & Exercise 53(10): 2101–2110. 10.1249/mss.0000000000002686. 33867501

[ejsc12206-bib-0007] Buist, I. , S. W. Bredeweg , K. A. P. M. Lemmink , W. van Mechelen , and R. L. Diercks . 2010. “Predictors of Running‐Related Injuries in Novice Runners Enrolled in a Systematic Training Program: A Prospective Cohort Study.” The American Journal of Sports Medicine 38(2): 273–280. 10.1177/0363546509347985. 19966104

[ejsc12206-bib-0008] Burke, A. , S. Dillon , S. O'Connor , E. F. Whyte , S. Gore , and K. A. Moran . 2023. “Aetiological Factors of Running‐Related Injuries: A 12 Month Prospective “Running Injury Surveillance Centre” (RISC) Study.” Sports Medicine ‐ Open 9(1): 46. 37310517 10.1186/s40798-023-00589-1 PMC10264338

[ejsc12206-bib-0009] Clarsen, B. , G. Myklebust , and R. Bahr . 2013. “Development and Validation of a New Method for the Registration of Overuse Injuries in Sports Injury Epidemiology: The Oslo Sports Trauma Research Centre (OSTRC) Overuse Injury Questionnaire.” British Journal of Sports Medicine 47(8): 495–502. 10.1136/bjsports-2012-091524. 23038786

[ejsc12206-bib-0010] Cuschieri, S. 2019. “The STROBE Guidelines.” Saudi Journal of Anaesthesia 13(Suppl 1): S31–s34. 10.4103/sja.sja_543_18. 30930717 PMC6398292

[ejsc12206-bib-0011] Desai, P. , J. Jungmalm , M. Börjesson , J. Karlsson , and S. Grau . 2021. “Recreational Runners with a History of Injury Are Twice as Likely to Sustain a Running‐Related Injury as Runners with No History of Injury: A 1‐Year Prospective Cohort Study.” Journal of Orthopaedic & Sports Physical Therapy 51(3): 144–150. 10.2519/jospt.2021.9673. 33356768

[ejsc12206-bib-0012] Herman, K. M. , W. M. Hopman , E. G. Vandenkerkhof , and M. W. Rosenberg . 2012. “Physical Activity, Body Mass Index, and Health‐Related Quality of Life in Canadian Adults.” Medicine & Science in Sports & Exercise 44(4): 625–636. 10.1249/mss.0b013e31823a90ae. 21971297

[ejsc12206-bib-0013] Hespanhol Junior, L. C. , L. O. Pena Costa , and A. D. Lopes . 2013. “Previous Injuries and Some Training Characteristics Predict Running‐Related Injuries in Recreational Runners: A Prospective Cohort Study.” Journal of Physiotherapy 59(4): 263–269. 10.1016/s1836-9553(13)70203-0. 24287220

[ejsc12206-bib-0014] Hespanhol Junior, L. C. , J. D. Pillay , W. van Mechelen , and E. Verhagen . 2015. “Meta‐Analyses of the Effects of Habitual Running on Indices of Health in Physically Inactive Adults.” Sports Medicine 45(10): 1455–1468. 10.1007/s40279-015-0359-y. 26178328 PMC4579257

[ejsc12206-bib-0015] Jungmalm, J. , M. L. Bertelsen , and R. O. Nielsen . 2020. “What Proportion of Athletes Sustained an Injury during a Prospective Study? Censored Observations Matter.” British Journal of Sports Medicine 54(2): 70–71. 10.1136/bjsports-2018-100440. 31113774

[ejsc12206-bib-0016] Kluitenberg, B. , M. van Middelkoop , D. W. Smits , E. Verhagen , F. Hartgens , R. Diercks , and H. van der Worp . 2015. “The NLstart2run Study: Incidence and Risk Factors of Running‐Related Injuries in Novice Runners.” Scandinavian Journal of Medicine & Science in Sports 25(5): e515–e523. 10.1111/sms.12346. 25438823

[ejsc12206-bib-0017] Lee, Y. C. , B. Lu , J. M. Bathon , J. A. Haythornthwaite , M. T. Smith , G. G. Page , and R. R. Edwards . 2011. “Pain Sensitivity and Pain Reactivity in Osteoarthritis.” Arthritis Care & Research 63(3): 320–327. 10.1002/acr.20373. 20957660 PMC3030930

[ejsc12206-bib-0018] Nielsen, R. , M. L. Bertelsen , D. Ramskov , C. Damsted , R. K. Brund , E. T. Parner , H. Sørensen , S. Rasmussen , and S. Kjærgaard . 2019. “The Garmin‐RUNSAFE Running Health Study on the Aetiology of Running‐Related Injuries: Rationale and Design of an 18‐month Prospective Cohort Study Including Runners Worldwide.” BMJ Open 9(9): e032627. 10.1136/bmjopen-2019-032627. PMC6731941 31494626

[ejsc12206-bib-0019] Nielsen, R. , E. T. Parner , E. A. Nohr , H. Sørensen , M. Lind , and S. Rasmussen . 2014. “Excessive Progression in Weekly Running Distance and Risk of Running‐Related Injuries: An Association Which Varies According to Type of Injury.” Journal of Orthopaedic & Sports Physical Therapy 44(10): 739–747. 10.2519/jospt.2014.5164. 25155475

[ejsc12206-bib-0020] Nielsen, R. , D. Ramskov , C. T. Blacket , and L. Malisoux . 2024. “Running‐Related Injuries Among More Than 7000 Runners in 87 Different Countries: The Garmin‐RUNSAFE Running Health Study.” Journal of Orthopaedic & Sports Physical Therapy 54(2): 1–9. 10.2519/jospt.2023.11959. 37970820

[ejsc12206-bib-0021] Nielsen, R. O. , M. L. Bertelsen , E. T. Parner , H. Sørensen , M. Lind , and S. Rasmussen . 2014. “Running More than Three Kilometers during the First Week of a Running Regimen May Be Associated with Increased Risk of Injury in Obese Novice Runners.” International Journal of Sports Physical Therapy 9(3): 338–345. 24944852 PMC4060311

[ejsc12206-bib-0022] Nygård Johansen, M. , S. Lundbye‐Christensen , and E. Thorlund Parner . 2020. “Regression Models Using Parametric Pseudo‐observations.” Statistics in Medicine 39(22): 2949–2961. 10.1002/sim.8586. 32519771

[ejsc12206-bib-0023] Pedisic, Z. , N. Shrestha , S. Kovalchik , E. Stamatakis , N. Liangruenrom , J. Grgic , S. Titze , S. J. H. Biddle , A. E. Bauman , and P. Oja . 2020. “Is Running Associated with a Lower Risk of All‐Cause, Cardiovascular and Cancer Mortality, and Is the More the Better? A Systematic Review and Meta‐Analysis.” British Journal of Sports Medicine 54(15): 898–905. 10.1136/bjsports-2018-100493. 31685526

[ejsc12206-bib-0024] van Gent, R. N. , D. Siem , M. van Middelkoop , A. G. van Os , S. M. Bierma‐Zeinstra , and B. W. Koes . 2007. “Incidence and Determinants of Lower Extremity Running Injuries in Long Distance Runners: A Systematic Review.” British Journal of Sports Medicine 41(8): 469–480. Discussion 480. 10.1136/bjsm.2006.033548. 17473005 PMC2465455

[ejsc12206-bib-0025] van Poppel, D. , M. van der Worp , A. Slabbekoorn , S. van den Heuvel , M. van Middelkoop , B. W. Koes , A. P. Verhagen , and G. Scholten‐Peeters . 2021. “Risk Factors for Overuse Injuries in Short‐ and Long‐Distance Running: A Systematic Review.” Journal of Sport and Health Science 10(1): 14–28. 32535271 10.1016/j.jshs.2020.06.006 PMC7856562

[ejsc12206-bib-0026] Videbæk, S. , A. M. Bueno , R. O. Nielsen , and S. Rasmussen . 2015. “Incidence of Running‐Related Injuries Per 1000 h of Running in Different Types of Runners: A Systematic Review and Meta‐Analysis.” Sports Medicine 45(7): 1017–1026. 10.1007/s40279-015-0333-8. 25951917 PMC4473093

[ejsc12206-bib-0027] Vincent, H. K. , J. E. 3rd Kilgore , C. Chen , M. Bruner , M. Horodyski , and K. R. Vincent . 2020. “Impact of Body Mass Index on Biomechanics of Recreational Runners.” PM & R 12(11): 1106–1112. 10.1002/pmrj.12335. 31994820

[ejsc12206-bib-0028] Yamato, T. P. , B. T. Saragiotto , and A. D. Lopes . 2015. “A Consensus Definition of Running‐Related Injury in Recreational Runners: A Modified Delphi Approach.” Journal of Orthopaedic & Sports Physical Therapy 45(5): 375–380. 10.2519/jospt.2015.5741. 25808527

