# Supplementary material for: Runners with a high body mass index and previous running‐related problems is a high‐risk population for sustaining a new running‐related injury: A 18‐month cohort study
Source: Eur J Sport Sci. 2024 Dec 10;25(1):e12206. doi: 10.1002/ejsc.12206 (PMC11680559; doi:10.1002/ejsc.12206)
Supplement: Supplementary file 1 — Table S1 [file EJSC-25-e12206-s001.docx]

| **Current fastest time at 5 km (baseline), n** |  |
| --- | --- |
| Below 14 min | 14(0.2) |
| 14-17 min | 102(1.5) |
| 17-20 min | 668(9.7) |
| 20-23 min | 1560(22.7) |
| 23-26 min | 1771(25.8) |
| 27-29 min | 1152(16.8) |
| 29-32 min | 667(9.7) |
| 32-35 min | 364(5.3) |
| Above 35 min | 348(5.1) |
| Uncertain | 215(3.1) |
| **Reasons for running, n(%)** |  |
| For wellbeing/health | 3952(57.6) |
| To get fitter | 923(13.5) |
| To compete | 918(13.4) |
| To get a break from work/family | 313(4.5) |
| To lose weight | 284(4.1) |
| As supplementary training | 267(3.9) |
| To socialize | 41(0.6) |
| Other | 142(2.1) |
| Uncertain | 7(0.1) |
| No response | 14(0.2) |

Supplementary material - The reasons for running in this study were mainly to sustain health
